# Social Technology: An Interdisciplinary Approach to Improving Care for Older Adults

**DOI:** 10.3389/fpubh.2021.729149

**Published:** 2021-12-09

**Authors:** Arthur Kleinman, Hongtu Chen, Sue E. Levkoff, Ann Forsyth, David E. Bloom, Winnie Yip, Tarun Khanna, Conor J. Walsh, David Perry, Ellen W. Seely, Anne S. Kleinman, Yan Zhang, Yuan Wang, Jun Jing, Tianshu Pan, Ning An, Zhenggang Bai, Jiexiu Wang, Qing Liu, Fawwaz Habbal

**Affiliations:** ^1^Department of Anthropology, Harvard University, Cambridge, MA, United States; ^2^Department of Global Health and Social Medicine, Harvard Medical School, Boston, MA, United States; ^3^Department of Psychiatry, Harvard Medical School, Boston, MA, United States; ^4^School of Social Work, University of South Carolina, Columbia, MO, United States; ^5^Graduate School of Design, Harvard University, Cambridge, MA, United States; ^6^School of Public Health, Harvard University, Boston, MA, United States; ^7^Harvard Business School, Boston, MA, United States; ^8^School of Engineering and Applied Sciences, Harvard University, Cambridge, MA, United States; ^9^Department of Medicine, Brigham and Women's Hospital and Harvard Medical School, Boston, MA, United States; ^10^Independent Researcher, New York, NY, United States; ^11^Department of Sociology, School of Social Sciences, Tsinghua University, Beijing, China; ^12^Institute of Anthropology and Ethnology, School of Social Development and Public Policy, Fudan University, Shanghai, China; ^13^School of Computer Science and Information Engineering, Hefei University of Technology, Hefei, China; ^14^Department of Sociology, School of Public Affairs, Nanjing University of Science and Technology, Nanjing, China; ^15^Policy Research Center, Ministry of Civil Affairs, Beijing, China; ^16^Jiangsu Industrial Technology Research Institute, Nanjing, China

**Keywords:** technology, social system, interdisciplinary, social integration, care, older adult

## Abstract

Population aging is a defining demographic reality of our era. It is associated with an increase in the societal burden of delivering care to older adults with chronic conditions or frailty. How to integrate global population aging and technology development to help address the growing demands for care facing many aging societies is both a challenge and an opportunity for innovation. We propose a social technology approach that promotes use of technologies to assist individuals, families, and communities to cope more effectively with the disabilities of older adults who can no longer live independently due to dementia, serious mental illness, and multiple chronic health problems. The main contributions of the social technology approach include: (1) fostering multidisciplinary collaboration among social scientists, engineers, and healthcare experts; (2) including ethical and humanistic standards in creating and evaluating innovations; (3) improving social systems through working with those who deliver, manage, and design older adult care services; (4) promoting social justice through social policy research and innovation, particularly for disadvantaged groups; (5) fostering social integration by creating age-friendly and intergenerational programs; and (6) seeking global benefit by identifying and generalizing best practices. As an emergent, experimental approach, social technology requires systematic evaluation in an iterative process to refine its relevance and uses in different local settings. By linking technological interventions to the social and cultural systems of older people, we aim to help technological advances become an organic part of the complex social world that supports and sustains care delivery to older adults in need.

## Social Technology: An Interdisciplinary Approach to Improving Care for Older Adults

Despite global volatility in economic, political, and health domains, two megatrends have persisted over recent years. One is population aging, which is becoming a global experience affecting virtually every nation in the world, with East and Southeast Asia undergoing the fastest increases (1). The demographic trend of aging, accompanied by increases in demands for care and by a continuous decrease in the numbers of working-age adults in most countries who can support older people, poses a serious threat to civil societies that seek to manage resources to meet the growing need for care. The other trend is the growth in the digital technology industry and an enduring belief in the need for technology among older people. Baby boomers indicate increasingly that their preference is to age in place at home. Equally clear is their hope to use technology to increase their ability to live independently (2).

The challenge is integrating these two mega trends so that each benefits. That is, technology can provide means to reduce the burden of care and increase quality of life for older adults and their caregivers, and the needs of the aging population can in turn stimulate targeted technology development and contribute to the much-expected arrival of a “silver economy”—an economic transformation that makes better use of older people's skills and knowledge and thereby contributes to a more availing future for them. To facilitate this integration, we have developed a research program based on an approach that we term *social technology*.

What do we mean by social technology? We begin by specifying what we do not mean. We do not mean to imply social engineering activities, such as implementing a centralized plan such as government-organized care practices that are the same for everyone in an effort to engineer people's lives (3). We are also not supporting the development of technologies for top-down social control through surveillance and security monitoring systems. And we definitely do not mean simply finding new ways to convince older people that they should accept a particular technological innovation.

Far from that, we aspire to integrate social science and engineering approaches in interdisciplinary methods that extend from planning through innovation, testing, and outcome assessment. We recognize that aging in this century is a challenge to systems that only systems' solutions can address through careful analysis and integration. We aim to build social technologies through a deliberate process of selection, development, and integration in various social and cultural settings. The goal is to create unique solutions that will assist individuals, families, and communities in coping more effectively with the disabilities of older persons by mitigating functional limitations caused by frailty and other sources of mobility problems that typically lead to isolation, cognitive decline, and depression. We will also address problems related to sensory losses, dementia, and mental illnesses, recognizing that these problems often overlap. We believe that social technologies can help reduce the burden on families and organizations that provide care to older adults who can no longer live independently.

Developing and identifying socially and culturally appropriate technological solutions to improve care for older persons with frailty and dementia is important; equally critical is integrating technology with social systems designed for the delivery of care—a complex process typically involving social, cultural, and ethical considerations. Effective social technology requires research efforts to integrate technological innovation with complex social systems to improve the quality of care and reduce the burden of caregiving for older people. Although the details of the social technologies have yet to be fully developed, we bring to this activity particular orientations and aspirations. Our vison is that, in practice, social technology would include these six specific considerations:

### Fostering Multidisciplinary Collaboration

Social technology starts with a comprehensive evaluation and understanding of problem scenarios and of many factors that potentially determine outcomes. By taking a biopsychosocial environmental perspective that acknowledges the complexity of human interactions within physical, social, and cultural contexts, we encourage multidisciplinary collaboration among social scientists (e.g., anthropologists, sociologists, psychologists, economists), engineers of different disciplines, and medical and public health researchers to maintain integrated views that are neither reductionistic nor inhuman. Multidisciplinary teams are essential to address older persons' needs. By using ethnography and other field research methods, we promote a bottom-up approach in which social scientists actively collect extensive information to understand the needs and living scenarios of older people, and their cultural and historical contexts, to guide the development of technological innovation or technology-based service innovation. The interactions and conversations among engineers and social scientists will optimize solutions and create new knowledge and innovations that are tailored appropriately. The policy framework within which care takes place will then increasingly tie together priorities, funding, and the local network of social services to health administration.

### Including Ethical and Humanistic Standards in Evaluation of Innovation

The value of an innovative solution to a care problem should be measured not by whether the technology employed departs from a previous technological method, but by how much the innovation improves the quality of care or its delivery and how compatible it is with local ethical standards and humanistic concerns. We particularly stress the importance of considering the perspectives of older persons and their families, emphasizing their desire for dignity and respect. Involving patients and family caregivers in this knowledge-generating process will be a conscious commitment, so that their input can help us create, modify, and improve social technology applications and prevent unintended negative consequences. This will also ensure the vital integration of cultural values and social requirements of the particular settings in all solutions. Moreover, we will encourage researchers to move away from being dispassionate experts and actively engage in the practice of care for others, making it “indispensable to the pursuit of social understanding” (4). The emotional and moral involvement of engineers will develop the appropriate appreciation of and thus sensitivity to older adults' needs and will enable innovations that serve that cause. Such social care, then, becomes integral to the research and to its outcome.

### Improving Social Systems

According to the social ecological model of care (5), older adult care is an integrated system that is ideally provided, supported, and sustained by stakeholders across several layers of a social system. These stakeholders typically include family caregivers who are usually the mainstay of care provided to older adults. Professional home care support programs, community-based daycare and exercise programs, long-term care facilities that provide an alternative to home care for older adults, and larger social policy agencies (e.g., insurance policy and housing policy agencies) that ensure sustainability of care are other parts of the system. We are committed to identifying and developing technological solutions that will be organically integrated within the care system and will assist, equip, strengthen, and honor family caregivers and other direct care workers. We will collaborate closely with those who deliver or manage care for older adults (e.g., nurses, physicians, pharmacists, physical and occupational therapists, psychotherapists, social workers, public health experts, economists, health and social service designers, and business executives) to improve service designs, perform feasibility testing, and optimize the use of supporting resources across physical and social distance. Social technologies will strengthen and integrate often fragmented care delivery systems. In some cases, a new social infrastructure (e.g., a community-based rehab device rental center) may need to be developed and/or additional training of personnel provided to facilitate and expedite the dissemination and diffusion of innovation.

### Promoting Social Justice Through Policy Research and Innovation

While aiming to bring technological solutions to most older adults, ensuring that socially disadvantaged groups be included as beneficiaries of technological solutions is critical. Special effort will be exerted in selecting and redesigning technologies toward a lower price range, so that ordinary people living in lower resource and marginal communities can afford them. We will collaborate closely with policy researchers and policy makers to explore social welfare policy innovations that will make technological solutions available for poor, marginal, and voiceless groups such as those in some disadvantaged rural areas or those with limited general and digital literacy.

### Fostering Social Integration

Among many benefits and impacts those technologies may bring to older adults, we particularly hope that technologies can contribute to socially cohesive and integrated societies. This means that technological solutions will help older persons increase their sense of social connection and reduce their feeling of loneliness and isolation. It could also mean that technologies will enable older people, in their post-retirement years, to attain their optimal functioning level so that they can continue to participate in social, learning, caregiving, and economic activities while maintaining and managing their physical and mental health. We envision that the available advancements in digital technologies, such as artificial intelligence and new imaging modalities, will be tailored to become part of social technologies and contribute to this vision. Similarly, technologies should increase opportunities for creating a truly intergenerational and age-friendly society in which active aging is an option and images of older persons as life-long learners and contributors gradually become part of a new social norm replacing the old prejudice of aging being associated with decline, deficiency, and dependency. Here social technology will contribute to reducing negative stereotypes and stigma.

### Seeking Global Benefit Through Best Practices

We hope that these solutions that combine technology with social science and sensitivity to cultural values will not only be useful in one location or one country but can also become generalized and applied in settings in many nations that also face the challenges of population aging and associated care challenges. By identifying and studying best practices of social technology that have been implemented in real-world settings, have also been evaluated systematically to obtain evidence of benefit, and are well-documented to improve transferability, we hope to contribute to the global effort to cope with the rapid growth of older adults' care needs and thereby strengthen the field of global aging.

### Final Thought

We treat social technology as emergent and experimental and as requiring systematic, iterative evaluation that refines its relevance and use in different local settings. We recognize that some of the concepts included in the social technology approach may have a history [e.g., (6, 7)]. Other concepts, such as gerontechnology and quality of life technology, may seem similar to social technology. Given that the emphasis in social technology is on the planning, community embeddedness, interpersonal and institutional consequences, and societal uses (and misuses) of technology, we view social technology as a system concept that includes gerontechnology and quality of life technology within its framework. By employing multidisciplinary collaboration and ethical/humanistic standards, we hope to enhance the process of innovation, and by emphasizing the importance of broader social benefits of technological solutions through improving care service, social justice, and social integration, we believe that this unified systems approach will help build a future society where intention and quality of care are more valued than efficiency and economic performance indicators.

Technologies will evolve as new inventions and innovations are developed and modified appropriately based on findings from social science investigations. At the same time, we recognize that technology may have unintended consequences. In the domain of healthcare an example is the electronic medical record, which has been criticized for making it more difficult for doctors to focus on patients rather than the computer and deliver more humanistic care (8). Despite this negative example, we still believe that technology has great potential for improving care and caregiving (9). By linking technological advances to the social and cultural systems of real-life of older persons, we aim to help technology become an organic part of the complex, dynamic social system that supports and sustains care delivery to older adults in need. In doing so, we intend to help build communities that are able to connect the benefits of technology to processes of care and that embrace serious concern for well-being and humanity (10).

## Data Availability Statement

The original contributions presented in the study are included in the article/supplementary material, further inquiries can be directed to the corresponding author.

## Author Contributions

AK, HC, FH, and SL are involved in the development of the idea of social technology and writing of the first draft. All other authors contributed to the elaboration of the concept and its specific significance in China as well as manuscript revision.

## Funding

The work was supported by the Jiangsu Industrial Technology Research Institute, China.

## Conflict of Interest

The authors declare that the research was conducted in the absence of any commercial or financial relationships that could be construed as a potential conflict of interest.

## Publisher's Note

All claims expressed in this article are solely those of the authors and do not necessarily represent those of their affiliated organizations, or those of the publisher, the editors and the reviewers. Any product that may be evaluated in this article, or claim that may be made by its manufacturer, is not guaranteed or endorsed by the publisher.
